# Role of Metabotropic Glutamate Receptors in Neurological Disorders

**DOI:** 10.3389/fnmol.2019.00020

**Published:** 2019-02-08

**Authors:** Rosalia Crupi, Daniela Impellizzeri, Salvatore Cuzzocrea

**Affiliations:** ^1^Department of Chemical, Biological, Pharmaceutical and Environmental Sciences, University of Messina, Messina, Italy; ^2^Department of Pharmacology and Physiology, Saint Louis University, St. Louis, MO, United States

**Keywords:** glutamate, metabotropic glutamate receptors, neurodegeneration, neuroinflammation, pain

## Abstract

Glutamate is a fundamental excitatory neurotransmitter in the mammalian central nervous system (CNS), playing key roles in memory, neuronal development, and synaptic plasticity. Moreover, excessive glutamate release has been implicated in neuronal cell death. There are both ionotropic and metabotropic glutamate receptors (mGluRs), the latter of which can be divided into eight subtypes and three subgroups based on homology sequence and their effects on cell signaling. Indeed, mGluRs exert fine control over glutamate activity by stimulating several cell-signaling pathways *via* the activation of G protein-coupled (GPC) or G protein-independent cell signaling. The involvement of specific mGluRs in different forms of synaptic plasticity suggests that modulation of mGluRs may aid in the treatment of cognitive impairments related to several neurodevelopmental/psychiatric disorders and neurodegenerative diseases, which are associated with a high economic and social burden. Preclinical and clinical data have shown that, in the CNS, mGluRs are able to modulate presynaptic neurotransmission by fine-tuning neuronal firing and neurotransmitter release in a dynamic, activity-dependent manner. Current studies on drugs that target mGluRs have identified promising, innovative pharmacological tools for the treatment of neurodegenerative and neuropsychiatric conditions, including chronic pain.

## Introduction

### Metabotropic Glutamate Receptors (mGluRs): Brain Distribution and Role in Neuroinflammatory and Neurodegenerative Diseases

Glutamate, a non-essential amino acid, is the main excitatory neurotransmitter of the central and peripheral nervous systems (CNS and PNS, respectively; Ferraguti et al., 2008). There are two major types of GluRs: ionotropic and metabotropic. Ionotropic glutamate receptors (iGluRs), such as N-methyl-d-aspartate (NMDA), amino-3-hydroxy-5-methyl-4-isoxazolepropionic acid (AMPA), and kainate receptors, are ligand-gated ion channels that stimulate fast excitatory neurotransmission (Dingledine et al., 1999). In contrast, metabotropic glutamate receptors (mGluRs) are G-protein coupled receptors (GPCRs) that have been categorized into three groups based on their signal transduction pathways and pharmacological profiles.

**Group I** metabotropic receptors, which include mGluR1 and mGluR5, are normally stimulatory and associated with phospholipase C activation and second messengers such as inositol and diacylglycerol production. **Group II** metabotropic receptors include mGluR2 and mGluR3, while **Group III** metabotropic receptors include mGluR4, mGluR6, mGluR7, and mGluR8. Both Group II and Group III receptors share a major sequence homology (~70%), and they normally inhibit glutamatergic neurotransmission (Conn and Pin, 1997). In addition, Group II and Group III metabotropic receptors are both negatively coupled to adenylyl cyclase. Elevated levels of mGluR1 have been reported in the neurons of the olfactory bulb, cerebellar cortex, ventral pallidum, globus pallidus, entopeduncular nucleus, lateral septum, magnocellular preoptic nucleus, and thalamic nuclei (Hubert et al., 2001). Their presence is also widespread in cerebellar Purkinje cells and in the mitral/tufted cells of the olfactory bulb. Moreover, notable Group I mGluR expression has been observed in the substantia nigra pars compacta (SNc), globus pallidus, lateral septum, and thalamic relay nuclei (Martin et al., 1992). In addition, the hypothalamus contains more mGlu1β receptors than mGlu1α receptors (Mateos et al., 1998). Previous studies have demonstrated that mGluR1 is associated with the postsynaptic specialization of excitatory synapses due to its subcellular localization, where it seems to be concentrated in the perisynaptic and extrasynaptic areas. Therefore, when concentrations of glutamate are elevated, excess glutamate leaks into the synaptic cleft, leading to activation of mGluRs. Several studies have investigated the role of mGluR1 in the cerebellar cortex, revealing that activation of such receptors is necessary for the stimulation of long-term depression (LTD) of excitatory neurotransmission at parallel Purkinje-fiber cellular synapses. Diacylglycerol is formed following activation of mGluR1; it is then split into 2-arachidonylglycerol (2-AG), the main endocannabinoid species of the CNS, by diacylglycerol lipase (Yoshida et al., 2006). Elevated mGluR5 expression has been observed in the telencephalon, particularly in the cerebral cortex, hippocampus, subiculum, nucleus accumbens, striatum, olfactory bulb, and lateral septal nucleus (Shigemoto et al., 1993; Romano et al., 1995). High expression of mGluR5 has also been observed in the superficial dorsal horn of the spinal cord (Berthele et al., 1999; Jia et al., 1999).

Among the Group II receptors, mGluR2 has been identified in only a few brain regions, such as the olfactory bulb and cerebellar cortex. In addition, mGluR2 is exclusively concentrated in neurons, primarily in the pre-terminal region of axons, far from the sites of neurotransmitter release (Tamaru et al., 2001). Presynaptic mGluR2/mGluR3 can be activated either by a surplus of synaptic glutamate or by glutamate released from astrocytes *via* the cystine–glutamate membrane antiporter (Kalivas, 2009). Modifications to the expression and activity of the cysteine–glutamate antiporter may influence the function of mGluR2 and mGluR3 in brain areas involved in drug dependence.

A key function of presynaptic mGluR2/mGluR3 is to reduce the release of neurotransmitters. Both receptor types are known to play a role in the modulation of synaptic plasticity, particularly in stimulating LTD of excitatory synaptic transmission (Grueter and Winder, 2005; Nicholls et al., 2006; Altinbilek and Manahan-Vaughan, 2009). A specific arrangement of synaptic plasticity has been observed in the mouse olfactory bulb, where stimulation of mGluR2 reduces gamma-aminobutyric acid (GABA)-ergic inhibition of mitral cells. Such inhibition enables the realization of a particular olfactory memory that closely reproduces the memory of the male pheromones that are produced during mating (Hayashi et al., 1993; Kaba et al., 1994). In the CNS, mGluR3 is extensively expressed in the olfactory tubercle, dentate gyrus, cerebral cortex, nucleus accumbens, lateral septal nucleus, striatum, amygdaloid nuclei, cerebellar cortex, and substantia nigra pars reticulata (Tanabe et al., 1993; Petralia et al., 1996; Tamaru et al., 2001). Expression of mGluR3 is observed presynaptically, postsynaptically, and on glial cells (Ohishi et al., 1993; Ferraguti and Shigemoto, 2006).

Group III mGluRs are expressed in the olfactory bulb, lateral reticular nucleus of the medulla oblongata, and pontine nuclei (Duvoisin et al., 1995; Saugstad et al., 1997; Corti et al., 1998). Several signaling pathways containing mitogen-activated protein kinases (MAPK) and PI3-kinase are coupled to the Group III mGluRs, allowing for control of synaptic transmission (Iacovelli et al., 2002, 2004). While mGluR7 is widely expressed in the brain, mGluR6 is not, instead exhibiting limited expression in the retina (Nakajima et al., 1993; Kinoshita et al., 1998). High mGluR7 expression has also been observed in the hippocampus, thalamus, neocortex, amygdala, hypothalamus, and locus coeruleus (Ngomba et al., 2011). Peripherally, mGluR7 is found in the adrenal glands, colon, and stomach, among other regions (Scaccianoce et al., 2003; Julio-Pieper et al., 2010).

Two other Group III receptors, mGlu4 and mGlu8, exhibit restricted expression in the brain (Pilc et al., 2008; Julio-Pieper et al., 2011). Although mGluR4 is primarily found in the cerebellum (Kinoshita et al., 1996; Shigemoto et al., 1997), it has also been observed in other areas, including the cerebral cortex, olfactory bulb, hippocampus, lateral septum, septofimbrial nucleus, striatum, thalamic nuclei, lateral mammillary nucleus, pontine nuclei, and dorsal horn (Fotuhi et al., 1994; Azkue et al., 2001; Corti et al., 2002). Moreover, previous studies have revealed that mGluR4 exhibits widespread peripheral expression in the gastrointestinal tract, pancreas, and adrenal glands (Chang et al., 2005; Sarría et al., 2006), and that such expression is highly concentrated around the active presynaptic area. While mGluR4, mGluR7, and mGluR8 are expressed in neurons, they are also expressed in oligodendrocyte precursor cells and recently formed oligodendrocytes.

Expression of mGluR8 in the CNS has been observed at the presynaptic level in the cerebellum, olfactory bulb, hippocampus, and cortical areas (Ferraguti and Shigemoto, 2006). However, mGluR8 expression has also been observed in peripheral tissues, such as the pancreas and testes (Julio-Pieper et al., 2011). Remarkably, previous studies have suggested that levels of mGluR8 are typically lower than those of mGluR4 and mGluR7 (Niswender and Conn, 2010).

Today, combined treatment approaches are the most attractive therapeutic strategies for numerous disorders, and several recent studies have highlighted the potential of multifunctional drug approaches (Kaiser and Nisenbaum, 2003). Because trauma and neurodegeneration in the CNS are influenced by several factors, multiple therapeutic approaches will likely be more effective than those directed at a single target. Neurons, astrocytes, microglia, oligodendrocytes, endothelial cells, and other circulating immune cells act in response to both acute and subacute injury and in chronic neurodegeneration. One multifunctional treatment strategy involves targeting mGluRs, which are expressed in several cell types commonly distributed throughout the CNS (Ferraguti and Shigemoto, 2006). Glial cells express both, ionotropic and mGluRs, as well as glutamate transporters. The different and heterogeneous locations of mGluRs in the CNS provide a promising opportunity to investigate drugs that selectively target different receptor subtypes. Several studies have demonstrated that mGluRs are expressed in lymphocytes as well as antigen-presenting cells, such as dendritic cells, microglia, and macrophages (Pacheco et al., 2006; Fallarino et al., 2010). In addition, mGlu5 and mGlu3 receptor activation can independently or cooperatively control several astrocyte functions, such as glutamate transporter activity, including astrocyte–arteriolar and astrocyte–neuronal interactions (Bradley and Challiss, 2012). In astrocytes, mGlu5 is the predominant or exclusive group I mGlu receptor subtype. Both mGluR3 and mGluR5 exert positive and negative influences on cell proliferation, and they are both highly expressed in cultured oligodendrocyte progenitor cells (Aronica et al., 2003). Moreover, mGluRs regulate cell migration, glutamate release, and the induction of the inflammatory phenotype in microglia (Barker-Haliski and White, 2015). Researchers have focused heavily on characterizing the involvement of mGluRs in various immune pathologies, including neuroinflammatory processes, in order to exploit them as novel targets for therapeutic strategies. Inflammatory events occur at different levels in the CNS, relative to those observed in other tissues. First, resident dendritic cells are absent in the CNS parenchyma, along with perivascular macrophages and vascular pericytes, which may shed light on the function of mature dendritic cells in the CNS. Second, stimulation of innate immune cells in the CNS parenchyma (e.g., astrocytes, microglia, and, in some regions, mast cells) may be reduced even under physiological conditions (Skaper et al., 2012). Moreover, the extravasation of immune cells and molecules towards the inflamed area—a process that is necessary for the activation of complement cascades and sustaining the immune response—is critical for the inflammatory response of the whole organism. Nevertheless, the blood–CNS barrier reduces the permeability of CNS microvessels, decreasing the magnitude of the inflammatory reaction. Only activated T-cells can penetrate the blood–CNS barrier, but they do not elicit an efficient (Patel et al., 2005) reaction to inflammation similar to that observed in peripheral tissues, where dendritic cells play a role in the adaptive immune response (Melchior et al., 2006). Consequently, the CNS reacts to inflammatory events when these events exert a direct effect on the CNS (i.e., in the case of pathogens and tissue damage, and when the inflammatory events are so severe that infiltrating T-cells are involved). In this way, neuroinflammation differs from inflammatory reactions that occur in other tissues, and can be thought to reflect the response of the CNS to altered homeostasis. This response is primarily mediated by the contribution of one or two cell systems: the glia of the CNS and the lymphocytes, monocytes, and macrophages of the hematopoietic system (Stoll and Jander, 1999). Neuroinflammation can be elicited by infection, autoimmunity, and toxins, but also by neurogenic factors such as noxious stimuli or psychological stress. However, extended neuroinflammation exceeds the limits of physiological control, resulting in harmful outcomes such as the stimulation of pro-inflammatory signaling pathways, increase doxidative stress, and the death of neighboring neurons. Neuroinflammation typically affects the severity and progression of neurodegenerative and psychiatric disorders, such as Alzheimer’s disease (AD), Parkinson’s disease (PD), Huntington’s disease (HD), multiple sclerosis (MS), stroke, and others (Lyman et al., 2014). Several cytokines such as interleukin-23 (IL-23), IL-12 (Bennett, 2013), IL-1b, and IL-6 affect neurodegenerative processes by attacking leukocytes, thus resolving neuroinflammation. Previous studies have demonstrated that different cell types such as oligodendrocytes, microglia, astrocytes (Domingues et al., 2016), both local and circulating lymphocytes, different dendritic cell subsets (Colton, 2013), and endothelial cells (Combes et al., 2012) are involved in neuroinflammation. Remarkably, several mGluR subtypes are expressed in these subtypes, both under stable conditions and during immune activation (Pacheco et al., 2007), suggesting that mGluRs play a role in regulating dissimilar immune responses in the CNS. The mechanisms by which mGluRs modulate immune responses are dependent on the specific subtype of mGluR that is implicated, and on the subset of targeted immune cells that bind the receptor. Normally, mGluR stimulation plays similar roles in the nervous and immune systems by theoretically responding to the negative effects of glutamate (Boldyrev et al., 2005). Indeed, mGluRs trigger widespread activation of various cell-signaling pathways in the CNS, suggesting that they are involved in some physiological and pathological processes associated with neurodegenerative disorders. Due to its potential roles in the pathophysiology of acute and chronic neurodegenerative diseases, glutamate has received much attention. Excess of glutamate is knowingly associated with excitotoxicity. Thus, the elevation in glutamate released from neuronal cells may induce acute neurodegeneration in both traumatic brain injury and cerebral ischemia (Ishikawa, 2013). Stimulation of AMPA, N-methyl-d-aspartate (NMDA), kainate, and Group I metabotropic receptors is involved in the neurotoxic processes underlying neurodegenerative diseases such as amyotrophic lateral sclerosis (ALS), motor neuron disease (MND), HD, AD, and PD. In status epilepticus, neuronal death is strictly correlated with NMDA receptor activation; whereas both, NMDA and AMPA receptors, are linked to the degeneration of neuronal tissue in cerebral ischemia.

## mGluRs in AD

AD is a progressive neurodegenerative disease that represents the main cause of dementia. Patients with AD experience memory loss associated with cognitive decline and motor fluctuations (Goedert and Spillantini, 2006). Due to increases in the number of older adults in the population, approximately 36 million people have been diagnosed with AD worldwide—a situation that is expected to double by 2050. Characterized by massive loss of synapses and neuronal death, AD affects approximately 10% of individuals over the age of 65, and approximately 40% of people over the age of 80 (Palop et al., 2006). AD progression disturbs brain areas involved in cognitive functions, such as the hippocampus, entorhinal and cerebral cortices, and ventral striatum. To date, treatments for AD provide provisional symptomatic relief only, and there is currently no cure or method for slowing disease progression. AD is associated with extracellular plaques that largely consist of beta-amyloid peptide (Aβ) aggregates (Glenner and Wong, 1984) and aberrantly phosphorylated tau, a microtubule-associated protein (Grundke-Iqbal et al., 1986). The protein Aβ, derived from amyloid precursor protein (APP) is the main constituent of the amyloid plaques. The need for drugs that can slow the progression of the pathological events that lead to synaptic dysfunction and neurodegeneration in AD is urgent. To date, the European Union (EU) has officially approved only four drugs for the treatment of AD (three cholinesterase inhibitors and memantine); however, none of these drugs has been shown to significantly modify disease activity. However, Group I mGluR agonists have demonstrated both neuroprotective and neurotoxic effects in *in vitro* and *in vivo* models of neurodegeneration (Nicoletti et al., 1999). Both Group II and Group III mGluRs are principally located at the levels of the presynaptic terminal in GABAergic and glutamatergic neuronal cells. Thus, activation of these receptors may decrease glutamate release (Cartmell and Schoepp, 2000). *In vivo* and *in vitro* studies have revealed that activation of Group III mGluRs exerts neuroprotective effects (Bruno et al., 2000). One *in vivo* study reported that treatment with low doses of the Group III mGluR agonist (+)-4-phosphonophenylglycine (PPG) exerts neuroprotective effects in wild-type mice, but not in mGluR4-knockout mice (Bruno et al., 2000). These results suggest that activation of mGluR4 is essential for neuroprotection. In addition, several studies have reported that Group III mGluR orthotropic agonists, such as L-AP4 and L-SOP, play neuroprotective roles in models of Aβ toxicity or excitotoxicity (Winkler et al., 1995; Bruno et al., 1996). In fact, numerous reports have indicated that discriminatory damage to cholinergic neurons in the basal forebrain (BF) is one of the most reliable modifications linked with AD at the initial stage of the disease (Winkler et al., 1995). Because it lowers NMDA levels, activation of mGluR7 protects BF neurons against such damage, thereby diminishing excitotoxicity (Gu et al., 2014). Furthermore, there is evidence that Aβ oligomers cause synaptotoxic effects at NMDA receptors (Malinow, 2012), which may represent the cause of cognitive dysfunction in AD. Despite such progress, precisely how mGluR signaling contributes to AD remains to be elucidated. Further *in vivo* studies using mGluR agonists and antagonists are required in order to determine whether targeting mGluRs is an effective pharmacological strategy for the treatment of AD. Such studies should aim to determine the role of mGluRs in various brain functions and neurological disorders in an effort to identify suitable treatment options.

## mGluRs in PD

PD represents the second most common neurodegenerative disease worldwide. The key feature of PD is selective loss of dopaminergic neurons in the SNc, leading to decreased dopamine levels in the striatum (Frisina et al., 2009). When degeneration surpasses 50%, diminished dopamine triggers the usual symptoms of the disease: postural instability, resting tremor, and hypokinesia (Lee and Liu, 2008). Moreover, degeneration of dopamine neurons in the SNc leads to increased glutamatergic activity in the subthalamic nucleus (STN), aggravating the motor symptoms of PD (Delong and Wichmann, 2015). Conventional PD therapy consists of the administration of 3,4-dihydroxyphenylalanine (L-DOPA), which aims to enhance motor function by increasing dopamine levels in the striatum (Schapira et al., 2006). While L-DOPA management is the gold-standard therapy for PD, chronic L-DOPA use is associated with a harmful “on-off” syndrome, a clinical state known as L-DOPA-provoked dyskinesia (LID; Lundblad et al., 2004). Recent studies have indicated that LID is caused by dysfunctional neuronal plasticity in the striatum due to the imbalance between glutamate and dopamine signaling (Picconi et al., 2012). Thus, targeting glutamate receptors may aid in the treatment of LID symptoms.

The first attempts to pharmacologically oppose glutamate hyperactivity involved the use of iGluR antagonists. While iGluRs exert adequate antiparkinsonian activity in preclinical models, they have been associated with debilitating side effects in humans, decreasing their applicability in clinical settings. Because of their modulatory role on glutamatergic transmission, mGluRs provide an alternative pathway for regulating increased glutamatergic transmission in the basal ganglia (Paoletti, 2011). Positive allosteric modulators (PAMs) of GluR4 have been proposed for the symptomatic management of PD. Stimulation of mGluR4 inhibits GABAergic discharge at synapses between striatal projection neurons and neurons of the globus pallidus (GPext), thus limiting the activity of the indirect pathway (Conn et al., 2005). Furthermore, mGlu4 PAMs have been shown to reduce motor symptoms in animal models of PD (Niswender and Conn, 2010). Interestingly, additional studies have demonstrated that mGlu4 PAMs exert protective effects against nigrostriatal damage induced by 1-methyl-4-phenyl-1,2,3,6-tetrahydropyridine (MPTP) in mice and 6-hydrxydopamine in rats (Betts et al., 2012). Therefore, mGluR4 PAMs may play a role as neuroprotective agents in PD. However, unlike mGluR5 PAMs, they are not believed to exert important therapeutic activity on LID (Ribeiro et al., 2014). Although selective mGlu5 receptor PAMs have been associated with antipsychotic activity, they may induce neurotoxicity in brain regions with high mGlu5 expression, such as the auditory cortex and hippocampus.

Recent research has indicated that the mGluR4 PAMVU0364770 enriches the motor response to a subthreshold dose of L-DOPA, but does not exert anti-dyskinetic activity (Iderberg et al., 2015). Another mGluR4 PAM, Lu AF21934, has been shown to reduce the incidence of LID, but not the severity. Using specific pharmacological tools, previous authors (Conn et al., 2005) reported that mGlu4 homodimers are presynaptically concentrated in the GPext, whereas mGlu2/mGlu4 heterodimers are expressed in the corticostriatal terminals. However, it remains to be determined whether the neuroprotective effects of mGluR4 PAMs, which are selective for homodimers, vary from those of PAMs that are selective for heterodimers. Adverse effects including dizziness and hallucinations have been reported, necessitating further clarification before clinical strategies can be developed. Additional research has indicated that mGlu2 and mGlu3 receptors do not exert beneficial effects on motor symptoms in animal models of PD: while the selective mGlu2/3 receptor agonist LY379268 improves rotarod performance in animal models of the disease, it does not modify akinesia in 6-OHDA-lesioned rats and may even worsen motor symptoms (Johnson et al., 2009). Group II mGluR agonists may be more appropriate for the treatment of neuropsychiatric symptoms associated with PD (Han et al., 2006).

Group III mGluRs (i.e., mGluR 4, mGluR7, and mGluR8) are expressed in GABAergic and glutamatergic terminals in the basal ganglia. Because these three mGluRs are expressed at the presynaptic level and are coupled to Gi/o, agonists or PAMs for such receptors can inhibit the release of both glutamate and GABA in PD. Moreover, Betts et al. (2012) revealed that allosteric potentiation of mGluR4 in the SNc diminished levels of inflammatory markers, improved motor deficits, and attenuated loss of dopaminergic neurons in a 6-OHDA rat model of PD. Several other studies have demonstrated that PAMs for this receptor can reverse both akinesia and catalytic disease caused by haloperidol, in addition to enhancing motor stimulation by L-DOPA in 6-OHDA-lesioned rats (Le Poul et al., 2012). Along with mGluR4, mGluR7 is particularly expressed at the level of the basal ganglia, where presynaptic actions at these receptors inhibit the release of glutamate. Thus, these receptors may represent targets for reducing excessive synaptic activation in PD. However, due to the dearth of selective ligands, the precise role of mGluR7 in PD remains unknown. To date, the first selective PAM for mGluR7 is AMN082, which exhibits modest antiparkinsonian effects (Mitsukawa et al., 2005). As with mGluR7, the lack of selective agonists for mGluR8 limits our ability to study its potential benefits in PD (Broadstock et al., 2012).Recent studies have demonstrated that the orthosteric agonist (S)-3, 4-dicarboxyphenylglycine (DCPG) exerts no influence in rodent models of PD (Broadstock et al., 2012). However, Johnson et al. (2013) reported that DCPG decreases haloperidol-induced catalepsy. The authors further reported that DCPG decreases reserpine-induced akinesia in a protracted, but not acute, 6-OHDA rodent model.

In summary, accumulating evidence suggests that targeting mGluRs can aid in managing motor symptoms and LID in PD. Thus, further preclinical and clinical studies that demonstrate the efficacy of agonists, antagonists, PAMs, and negative allosteric modulators (NAMs) for all mGluR types are critical in advancing therapeutic strategies for PD.

## mGluRs in HD

HD is an autosomal dominant neurodegenerative disease associated with the presence of polyglutamine, which is localized in the amino-terminal region of the huntingtin protein (htt). Specifically, this pathology is represented by a single genetic mutation that promotes the development of the disease in animal models with genetic modifications that summarize the traits of HD (Pouladi et al., 2013). HD is associated with several symptoms, including the loss of cognitive function, involuntary body movements and chorea, psychiatric disturbances, and death (Li and Li, 2004). Among the possible mechanisms, many studies have focused on the mutation of htt as the cause of gradual neuron loss in the neocortical regions and caudate-putamen in patients with HD.

Previous studies have indicated that inhibition of presynaptic glutamate release *via* activation of both Group II and Group III mGluRs may attenuate the processes associated with excitotoxicity in patients with HD. Group II and Group III mGluRs, particularly those positioned at corticostriatal presynaptic terminals, can mediate negative feedback control for glutamate release (Calabresi et al., 1999). Treatment with LY379268 (1.2 mg/kg VO) has been reported to increase survival time and decrease early pathological hyperactivity in a transgenic mouse model of HD (e.g., R6/2 mouse); however, such treatment does not improve rotarod performance or htt intranuclear inclusions (Schiefer et al., 2004). However, subcutaneous administration of LY379268 at 20 mg/kg was associated with positive effects in R6/2 mice, including increased survival time, improved rotarod performance, normalization of locomotor performance, and a 20% decrease in neuronal loss in both the cortex and striatum. Nevertheless, LY379268 was unable to modify the frequency or size of htt aggregates (Schiefer et al., 2004). Another study reported that brain-derived neurotrophic factor (BDNF) expression increases in layer 5 of the motor cortex following administration of LY379268, indicating that activation of mGluR2/3 may counteract neuronal cell death by increasing or diminishing levels of BDNF.

In summary, activation of mGluR2/mGluR3 may counteract the release of glutamate and diminish excitotoxicity. However, the roles of mGluR1/mGluR5 in regulating neuronal death remain to be clarified. Partial activation of these receptors, as well as activation of mGluR2/mGluR3, excites neuroprotective cell-signaling pathways, stimulating increases in BDNF expression. Such effects may be associated with improvements in the symptoms of HD (Li and Li, 2004).

## mGluRs in Chronic Stress-Related Disorders

Depression and anxiety are psychiatric conditions related to chronic stress, and they are classified as important public health issues. The etiologies of these disorders are complex, and psychosocial stressors are among the most debated risk factors. Considering the significance of glutamate in the brain, pharmacological interventions for these disorders should target excesses in glutamate transmission while leaving normal glutamatergic transmission unaltered. Pharmacological modulation of mGluR subtypes may allow for such modification (Bergink et al., 2004). The functional diversity and distribution of the different mGluR subtypes may allow for selective targeting of individual receptor subtypes, which may in turn lead to the development of novel strategies for the treatment of emotional disorders. Preclinical data have suggested that ligands for mGluR subtypes can aid in the management of mood disorders such as depression and anxiety. Furthermore, selective mGluR ligands have begun to show promise in clinical trials, with some compounds exhibiting outstanding clinical efficacy.

Among the stress-related psychiatric conditions, major depressive disorder (MDD), anxiety, and drug abuse are significant health concerns worldwide (Cryan and Holmes, 2005). These pathologies are very complex, and chronic psychosocial stressors have been recognized as the greatest risk factors associated with these conditions (Cryan and Holmes, 2005). Research has revealed strong comorbidity between depression/mood disorders and anxiety (Cortese and Phan, 2005): approximately half of patients with anxiety also meet the criteria for MDD. Both disorders are characterized by disproportionate excitability within crucial brain circuits. The L-glutamate system, considered the primary excitatory neurotransmitter system within the circuits of emotion and cognition, plays a prominent role in the etiopathology and persistence of disorders related to mental health. One study using human data has linked dysfunction in the L-glutamate system to the pathogenesis of psychiatric conditions (Cortese and Phan, 2005). In fact, alterations in glutamate levels have been found in the cerebrospinal fluid (CSF), plasma, and brains of patients with mood and anxiety disorders. Postmortem findings have confirmed these data, revealing that patients with depression and bipolar disorder exhibit substantial increases in glutamate levels in both the frontal and dorsolateral prefrontal cortex, respectively (Lan et al., 2009). Moreover, clinical neuroimaging data have consistently reported volumetric alterations in brain areas where glutamatergic neurons predominate, such as the amygdala, hippocampus, and numerous cortical regions (Lorenzetti et al., 2009). In addition, expression of mGlu2Rs in the hippocampus has been strongly correlated with the mechanisms underlying resilience (or non-resilience) to stress, which lie at the core of the pathophysiology of MDD and other stress-related disorders (McEwen et al., 2015).

## mGluRs in the Physiology of Stress

Group III mGluRs have received less attention than Group I and Group II mGluRs, likely due to the lack of selective and brain-penetrant pharmacological tools (Semyanov and Kullmann, 2000). However, Group III mGluRs are thought to be implicated in several psychological disorders and physiological conditions due to their role in regulating glutamatergic and GABAergic neurotransmission (Semyanov and Kullmann, 2000). Domin et al. (2014) demonstrated that intraventricular injection of the Group III mGluR agonist (1S,3R,4S)-1-aminocyclopentane-1,3,4-tricarboxylic acid (ACPT-I) can induce antidepressant- and anxiolytic-like effects. In particular, such anxiolytic effects have been observed in stress-induced hyperthermia (SIH), elevated plus maze (EPM) and Vogel conflict tests. Moreover, the antidepressant-like effects of ACPT-I have been observed in mice subjected to the forced swim test (FST; Domin et al., 2014). Klak et al. (2007) reported that combined treatment with the mGlu4-selective PAM 7-hydroxyimino-N-phenyl-1,7 adihydrocyclopropa[b]chromene-1a-carboxamide (PHCCC) and a non-effective dose of ACPT-1 exerts antidepressant-like effects. Subsequent studies have demonstrated that local ministration of PHCCC to the basolateral amygdala exerts dose-dependent anti-conflict effects in rats subjected to the Vogel conflict test. These results indicate that positive allosteric modulation of mGlu4Rs may represent an alternative therapeutic strategy for the treatment of state anxiety (Stachowicz et al., 2004).

Recent data have further revealed that (1R,2S)-2-[(3,5-dichlorophenyl)carbamoyl]cyclohexane-1-carboxylic acid (VU0155041), an mGlu4 PAM, exerts anxiolytic effects in animals subjected to the elevated-zero maze. Furthermore, the novel mGlu4 PAMs (1S,2R)-2-[(aminooxy)methyl]-N-(3,4-dichlorophenyl)cyclohexane-1-carboxamide (Lu AF21934) and 4-methyl-N-[5-methyl-4-(1H-pyrazol-4-yl)-1,3-thiazol-2-yl]pyrimidin-2-amine (ADX88178) have been shown to promote anxiolytic effects in acute rodent models [e.g., stress-induced hyperthermia (SIH), four-plate test (FPT) and marble-burying test (MBT)], and to be effective in several PD models (Kalinichev et al., 2014). Remarkably and reciprocally, mGlu4-deficient mice exhibit increased anxiety in acute models (i.e., open-field text and elevated-zero maze), as well as decreased sensorimotor function in the rotarod test. Such data suggest that mice exhibited improvements in amygdala-dependent cued-fear conditioning (Davis et al., 2013). In accordance with these results, additional studies have reported that mice lacking mGluR8 exhibit higher levels of anxiety than control animals. Moreover, mice exposed to new, aversive environments exhibited greater neuronal activation in stress-related brain areas (Linden et al., 2003). These data suggest that mice deficient in mGluR4 or mGluR8 exhibit improved reactivity to stressors.

The stimulation of mGluR8 with the selective agonist (S)-3, 4-DCPG diminishes innate anxiety levels in the open-field and EPM tests, as well as the expression of contextual fear, without disturbing processes associated with cued fear (Fendt et al., 2013). An mGlu8 receptor-preferring agonist, 2-amino-2-(4-phosphonophenyl)acetic acid (RS-PPG), provokes dose-dependent antidepressant-like effects in the FST following central administration, while the mGlu8-selective PAM 2-[(4-bromophenyl)methyl-sulfanyl]-N-(4-butan-2-ylphenyl) acetamide (AZ12216052) decreases levels of anxiety in both the EPM and open-field tests (Duvoisin et al., 2010). Over the last several decades, preclinical and clinical studies have provided encouraging data, revealing that the glutamatergic system of the brain plays a key role in the physiology of psychiatric disorders. Unfortunately, very few studies have investigated the contribution of the glutamatergic system to the pathophysiology of chronic psychosocial stress (Hammen, 2005). For these reasons, current drug discovery efforts targeting mGluRs have focused on identifying pharmacological agents that can effectively treat psychiatric disorders (Mercier and Lodge, 2014).

## mGluRs and Pain

Due to the concurrence of different types of pain, chronic pain is a complex syndrome that remains difficult to treat. Nevertheless, mGluRs may represent suitable targets for counteracting the nociceptive and persistent forms of pain. Previous studies have established the role of mGluRs, expressed at peripheral and brain area associated with pain modulation, in efficiently reducing pain hypersensitivity. Such studies have demonstrated that mGluRs control the perception of physiological pain, and that they are associated with the development of peripheral and central pain (Chiechio and Nicoletti, 2012).

Over the last several decades, research has demonstrated that mGluRs represent promising targets in the treatment of chronic pain. For example, these studies have revealed that pain hypersensitivity is efficiently controlled be either blocking Group I mGluRs or activating both Group II and III mGluRs. These effects can be achieved using orthotropic ligands that can block or activate specific mGluR subtypes, or using allosteric ligands that positively (PAM) or negatively (NAM) regulate mGluR functions (Govea et al., 2012). In this review article, we focus on the role of Group III mGluRs in the treatment of chronic pain.

Group III mGluRs are expressed throughout the pain neuraxis, from the peripheral nerves to the CNS. Research has indicated that mGluR8 is expressed in unmyelinated fibers of the digital nerves, where they adversely modify the activity of transient receptor potential vanilloid 1 (TRPV1) receptors on nociceptors by inhibiting the activity of adenylyl cyclase (Govea et al., 2012). Several studies have also demonstrated that intraplantar injection of a Group III mGluRs agonists, such as L-AP4, diminishes the hyperalgesia triggered by the TRPV1 agonist capsaicin (Govea et al., 2012). Similar to Group II mGluRs, peripheral Group III mGluRs have been implicated in the management of hyperalgesia after inflammatory states. In one study, *in situ* treatment with L-AP4, a Group III mGluR agonist, reduced hyperalgesia in a carrageenan-induced model of arthritic pain in the knee joint (Lee et al., 2013). Moreover, stimulation of Group III mGluRs in the dorsal horn of the spinal cord reduces the shooting pain sensation generated by second-order neurons by monitoring excess glutamatergic transmission in models of both neuropathic and inflammatory pain (Zhang et al., 2009).

Then GluR4 subtype is expressed on the presynaptic terminals of C-fibers and spinal neuron terminals in inner laminae II of the dorsal horn. Vilar et al. (2013) reported that stimulation of mGluR4 in the dorsal horn inhibits the development of both neuropathic and inflammatory pain by decreasing glutamatergic transmission. The mGluR7 subtype has also been discovered in the presynaptic terminals of sensory neurons in laminae I and laminae II of the dorsal horn. Interestingly, mGluR7 does not appear to play a relevant role in chronic pain, as intrathecal administration of mGluR7 PAMs does not diminish hyperalgesia in a model of neuropathic pain (Wang et al., 2011). Moreover, previous studies have revealed that Group III mGluRs are significantly expressed in the supraspinal region in conditions associated with pain. Nevertheless, mGluR7 and mGluR8 appear to exert opposing effects in the periaqueductal gray (PAG), amygdala, and rostral ventromedial medulla (RVM). Many authors have reported that systemic activation of both mGluR7 and mGluR8 is efficient in decreasing neuropathic and inflammatory pain; moreover, local stimulation of mGluR7 within the PAG and amygdala intensifies pain, while mGluR7 blockade reduces both inflammatory and neuropathic pain. Stimulation of mGluR8 in the amygdala, PAG, dorsal striatum, and RVM has also been reported to reduce inflammatory and neuropathic pain (Marabese et al., 2007b). Taken together, these findings indicate that Group III mGluR ligands may inhibit pain hypersensitivity in patients with chronic pain. These results are particularly significant for mGluR4, since activation of such receptors in the spinal cord decreases the perception of chronic pain without affecting normal pain perception.

Few studies have investigated the impact of systemic administration of Group III mGluR agonists or antagonists on pain management. Systemic administration of the mGlu8 receptor agonist (S)-3,4-DCPG results in formalin-induced nocifensive behaviors in models of carrageenan-induced mechanical allodynia and thermal hyperalgesia, and in the first stage of neuropathic pain (Marabese et al., 2007a). Research regarding selective mGluR7 NAMs has revealed that mGluR7 plays a key role in physiological and pathological pain conditions. *In vivo* studies of the mGluR7 NAM 6-(4-methoxyphenyl)-5-methyl-3-pyridinyl-4-isoxazolo[4,5-c]pyridin-4(5H)-one (MMPIP) have reported that negative allosteric alteration of the mGluR7 worsens cognitive performance in both radial arm maze tasks and object recognition tests, in addition to reducing social interaction. Additional studies have indicated that MMPIP does not affect depression- or anxiety-like behaviors, motor coordination, sensorimotor gating, seizure threshold, or nociception in healthy rats and mice (Hikichi et al., 2010). Subcutaneous administration of MMPIP has been found to trigger sensory and affective/cognitive symptoms of neuropathic pain in a spared nerve injury model of neuropathic pain, although such treatment exerted no effect in control mice. Palazzo et al. (2015) demonstrated that alterations in receptor expression in supraspinal areas such as the dorsal raphe, basolateral amygdala, PAG, hippocampus, and prelimbic cortex—which are observed in neuropathic pain—may be required for MMPIP efficacy. Among the selective mGluR7 NAMs, 7-hydroxy-3-(4-iodophenoxy)-4H-chromen-4-one (XAP044) inhibits long-term potentiation in brain tissues enclosing the lateral amygdala in wild-type mice, but not in mGluR7-knockout mice, suggestive of XAP044-specific functions for mGluR7 (Gee et al., 2014). Palazzo et al. (2015) further demonstrated that subcutaneous treatment with XAP044 can modify both mechanical allodynia and anxiety- and depression-like behaviors in mice with neuropathic pain. Subcutaneous administration of another selective mGluR7 NAM, (**+**)-6-(2,4-dimethylphenyl)-2-ethyl-6,7-dihydrobenzo [d]oxazol-4(5H)-one (ADX71743), in rodents leads to anxiolytic-like effects in the EPM and MBTs. This selective mGluR7 NAM has been reported to decrease amphetamine-provoked hyperactivity without altering locomotor activity under physiological conditions (Kalinichev et al., 2013).

## Conclusion

Knowledge regarding mGluRs has improved exponentially over the last several years. Research has uncovered new mechanisms of action and ligands for these receptors, which may allow for the development of novel therapeutic strategies for neuroinflammatory diseases associated with brain excitability. The discovery of new compounds has encouraged the development of selective tools, some of which can be implemented in clinical practice. Moreover, the diversity and heterogeneous distribution of mGluR subtypes in the brain may allow for the targeting of specific mGluR subtypes implicated in different functions of the CNS, which may in turn aid in the development of novel treatment strategies for psychiatric and neurological disorders, including depression, anxiety, chronic pain, AD, and PD.

Treatments targeting iGluRs in the CNS have failed due to multiple side effects, including cognitive and motor impairment. In general, targeting glutamatergic neurotransmission *via* the modulation of mGluRs holds great promise for the management of several CNS diseases, with the potential for fewer side effects. Drugs targeting mGluRs achieve their therapeutic effects by reducing excitatory drive, either *via* antagonism of Group I mGluRs or activation of Group II and III mGluRs (one exception is represented by the use of mGluR5 PAMs for the management of cognitive deficits linked with schizophrenia).

In clinical practice, the results of targeting mGluRs will depend on whether the targeted glutamatergic pathways are directly or indirectly linked to the pathological condition of interest. Thus, clinical efficacy may depend on indirect potentiation of GABAergic, dopaminergic, or other neurotransmitter systems. Novel therapeutic approaches may benefit from selective targeting of multiple mGluRs. For example, mixed Group I antagonism/Group II or III agonism may be the key to developing effective treatment strategies for some disorders. However, few clinical studies have supported the therapeutic benefit of mGluR modulation in the management of psychiatric and neurological disorders, with the exception of a positive clinical trial for the mGluR2/3 agonist LY2140023 in the treatment of schizophrenia. Further clinical studies are required to determine whether targeting of multiple mGluRs can be effective in human patients with neuropsychiatric and neurological disorders.

## Author Contributions

RC and DI wrote this review article. SC edited and revised it.

## Conflict of Interest Statement

The authors declare that the research was conducted in the absence of any commercial or financial relationships that could be construed as a potential conflict of interest.
